# *Capnocytophaga gingivalis* and Oral Squamous Cell Carcinoma: Novel Insight from Periodontitis Patients

**DOI:** 10.3390/microorganisms14071535

**Published:** 2026-07-14

**Authors:** Uros Tomic, Sanja Petrovic, Djordje Mihailovic, Jelena Carkic, Nadja Nikolic, Jelena Milasin, Ana Pucar

**Affiliations:** 1 Clinic for Periodontology and Oral Medicine, School of Dental Medicine, University of Belgrade, 11000 Belgrade, Serbia; uros.tomic@stomf.bg.ac.rs; 2 Laboratory for Microbiology, School of Dental Medicine, University of Belgrade, 11000 Belgrade, Serbia; sanja.petrovic@stomf.bg.ac.rs; 3 Department of Dentistry, Faculty of Medical Sciences Pristina, University of Pristina, 38220 Kosovska Mitrovica, Serbia; drdjordjemihailovic@gmail.com; 4 Scientific Laboratories, Implant-Research Center, School of Dental Medicine, University of Belgrade, 11000 Belgrade, Serbia; jelena.carkic@stomf.bg.ac.rs (J.C.); jelena.milasin@stomf.bg.ac.rs (J.M.)

**Keywords:** oral squamous cell carcinoma, *C. gingivalis*, periodontitis, *qPCR*, carcinogenesis

## Abstract

Background: Oral squamous cell carcinoma (OSCC) has increasingly been associated with oral microbiota and chronic periodontal inflammation. While major periodontal pathogens have been extensively studied, the role of *Capnocytophaga gingivalis* (*C. gingivalis*) in oral carcinogenesis remains unclear. This study investigated the prevalence and quantified the presence of *C. gingivalis* in benign oral lesions, oral potentially malignant disorders (OPMDs), and OSCCs, as well as its association with carcinogenesis-related gene expression. Methods: Ninety patients with periodontitis were included: 30 with benign lesions, 30 with OPMDs, and 30 with OSCCs. *C. gingivalis* quantification was performed using qPCR, while relative expression of *VEGF*, *Cyclin D1*, *PIK3CA*, *DUSP16*, *mTOR*, and *MAPK14* was analyzed by RT-qPCR. Results: *C. gingivalis* was detected in 2 benign lesions, 11 OPMDs, and 18 OSCC samples (*p* < 0.001). Overall bacterial burden was significantly higher in OPMD and OSCC groups compared to benign lesions (*p* = 0.001). Expression of *PIK3CA* and *MAPK14* was significantly increased in the OPMD and OSCC groups. In OSCC samples, *C. gingivalis* abundance positively correlated with *VEGF* and *Cyclin D1* expression. Conclusions: *C. gingivalis* showed progressively increased prevalence and abundance across examined lesions and was associated with altered expression of genes involved in carcinogenesis, supporting its potential role in OSCC progression.

## 1. Introduction

For many years, the risk factors for oral squamous cell carcinoma (OSCC) were considered well established, with smoking and alcohol consumption identified as the most prominent [1,2,3]. However, cases of OSCC have also been reported in non-smokers and individuals who do not consume alcohol [4], pointing to the necessity of finding novel explanations for cancer initiation. Since then, additional factors have emerged as potential contributors to OSCC, including immunodeficiencies, such as HIV infection [5], exposure to different substances [6,7], and dietary practices [8] have emerged as potential risk factors. Among infectious agents, human papillomavirus (HPV), particularly its oncogenic types, was one of the first to be associated with OSCC [9]. Increasing evidence also highlights the contribution of oral hygiene habits and oral microbiota to the etiopathogenesis of OSCC. The majority of studies exploring the association between oral microorganisms and OSCC have focused on key periodontal pathogens belonging to the red and orange Socransky complexes, including *Porphyromonas gingivalis*, *Fusobacterium nucleatum*, and *Treponema denticola* [10,11,12] as well as *Candida albicans* [13].

Conversely, the bacterium *Capnocytophaga gingivalis* (*C. gingivalis*), classified within the Socransky green complex and recognized as a secondary colonizer in dental biofilm formation [14], remains underexplored in relation to oral carcinogenesis, leaving a gap in the understanding of its potential role in OSCC.

Studies investigating *C. gingivalis* in the context of periodontal health have primarily focused on its prevalence in healthy versus diseased periodontium, without addressing potential differences in virulence factors between strains associated with these conditions [15].

Since periodontitis is a polymicrobial disease driven by the interactions of multiple microbial species [16], *C. gingivalis* is unlikely to function independently but rather as part of that complex microbial community, which shapes its virulence and biological impact. The virulence of *C. gingivalis* may vary considerably, manifesting differently in healthy periodontium compared to the periodontitis environment. Yet, how these context-dependent dynamics influence its pathogenic potential remains poorly understood.

*C. gingivalis*, as already stated, has generally been overlooked as a potential etiological agent in OSCC. One study reported that the presence of *C. gingivalis* in saliva, together with other periodontal bacteria, might serve as a diagnostic marker for OSCC [17], while another showed its presence in OSCC tissue specimens and proposed a possible role in modulating the tumor microenvironment [18].

Zhang et al. proposed three principal mechanisms by which oral microbiota may contribute to OSCC: chronic inflammation, anti-apoptotic activity, and direct production of carcinogenic substances [19]. To date, no direct evidence has linked *C. gingivalis* to these mechanisms. However, given that *C. gingivalis* shares certain virulence factors with established periodontal pathogens, it is conceivable that it may contribute to OSCC pathogenesis through chronic inflammation and anti-apoptotic activity.

One of the key anti-apoptotic pathways is the PI3K/AKT/mTOR signaling pathway, which transduces extracellular signals and regulates cell growth, proliferation, metabolism, angiogenesis, etc. Its role in carcinogenesis is well established, making it a target for anticancer therapy [20,21]. AKT activation also promotes Cyclin D1 (CCND1) expression, thereby facilitating the transition from the G1 to the S phase of the cell cycle. Increased Cyclin D1 expression has been implicated in carcinogenesis, particularly in OSCC [22].

Another key signaling pathway with a context-dependent role in carcinogenesis is the mitogen-activated protein kinase (MAPK) pathway. The MAPK14 (p38 MAPK) pathway plays a critical role in regulating cell differentiation, apoptosis, and autophagy (23). Depending on the biological context and tumor stage, p38 MAPK signaling may exert either tumor-suppressive or tumor-promoting effects. In early stages of carcinogenesis, it has been associated with cell cycle arrest and apoptosis, whereas in advanced stages, principally in the presence of chronic inflammation, it may help tumor invasion, metastasis, and angiogenesis (23). MAPK overexpression has been reported in more than 50% of OSCC cases (24). MAPK activity can be regulated by dual-specificity phosphatases (DUSPs) [23], among which DUSP16 acts as a negative regulator of JNK and p38 MAPK signaling [23]. Dysregulation of DUSP16 has been linked to carcinoma progression, mostly through its disruption of MAPK signaling pathways [24]. In addition to promoting cell survival, angiogenesis is a hallmark of tumor progression. Vascular endothelial growth factor (VEGF), a family of signaling proteins, is one of the chief mediators of angiogenesis, and its overexpression has been well established in OSCC [25,26].

The activity of these signaling pathways is under the influence of multiple factors, including infections. Numerous studies have demonstrated that systemic (non-oral) pathogens such as *Staphylococcus aureus*, *Streptococcus pneumoniae*, *Chlamydia pneumoniae*, *Helicobacter pylori*, HPV, and Epstein–Barr virus (EBV) can modulate these pathways [27,28,29,30,31,32,33]. Besides non-oral microorganisms, some oral microorganisms, including *P. gingivalis*, *Prevotella intermedia*, *Porphyromonas endodontalis*, *F. nucleatum*, *Veillonella parvula*, and *Aggregatibacter actinomycetemcomitans*, have been shown to impact these signaling cascades [34,35,36,37,38,39,40].

Taken together, available evidence indicates that the role of *C. gingivalis* in OSCC remains poorly understood. No study has directly examined whether its abundance is associated with the expression of key genes involved in carcinogenesis. Indirect evidence proposes that, through virulence-associated mechanisms, *C. gingivalis* may modify these processes, principally in patients with periodontitis, where the polymicrobial environment stimulates virulence factor expression.

To address this knowledge gap, the present study focused exclusively on *C. gingivalis* as a candidate microorganism and did not aim to characterize the overall oral microbial community or compare the abundance of other periodontal pathogens. This reductionist approach was intentionally adopted to evaluate whether *C. gingivalis* alone shows an association with host gene expression before addressing more complex polymicrobial interactions. Therefore, the goal of this study was to detect and quantify *C. gingivalis* in OSCC tissue specimens and to compare its abundance among OSCC, benign lesions, and potentially malignant oral disorders in patients with periodontitis. Additionally, the study intended to evaluate whether the bacterial load of *C. gingivalis* correlates with the expression of key genes and signaling pathways involved in carcinogenesis, inflammation, proliferation, apoptosis, and angiogenesis, including *VEGF*, *Cyclin D1*, *PIK3CA*, *DUSP16*, *mTOR*, and *MAPK14*. Gene expression patterns of these markers were also compared across the examined groups.

## 2. Materials and Methods

### 2.1. Participants and Study Design

This cross-sectional study was conducted from January 2022 to December 2024. The study protocol received approval from the Ethical Committee of the School of Dental Medicine, University of Belgrade (No. 36/7). All procedures were performed in accordance with the ethical and scientific principles of the Declaration of Helsinki.

The study included patients referred for evaluation, diagnosis, and potential treatment of suspicious oral mucosal lesions (benign lesions, oral potentially malignant disorders, or oral squamous cell carcinomas) at the Department of Oral Medicine and Periodontology and the Department of Maxillofacial Surgery, School of Dental Medicine, University of Belgrade. Patients were comprehensively briefed on the trial and its objectives and risks, and they provided written informed consent before being enrolled in the study.

The inclusion criteria were as follows: (1) age ≥ 25 years; (2) presence of a suspicious oral mucosal lesion; and (3) diagnosed periodontal disease (periodontitis) with a minimum of 15 remaining teeth. The exclusion criteria were as follows: (1) periodontal treatment involving antibiotics, antiseptics, or anti-plaque mouthwashes within the previous two months; (2) existence of dentures; (3) history of radiotherapy and/or chemotherapy in the head and neck region; and (4) previously performed biopsies of the suspicious lesion. A total of 90 patients fulfilled the defined criteria, and their complete anamnestic data were collected and documented in standardized medical records.

A full head and neck examination was performed, accompanied by a visual inspection of the oral cavity during clinical assessment.

Periodontal parameters, including periodontal probing depth (PPD), clinical attachment level (CAL), and gingival recession (GR), were measured at six sites surrounding each existing tooth (mesio-buccal, mid-buccal, disto-buccal, mesio-lingual, mid-lingual, and disto-lingual) using a periodontal probe (North Carolina–Hu-Friedy, Chicago, IL, USA). Oral hygiene status was evaluated using the plaque index (PI), and inflammation was assessed using the bleeding on probing index (BOP) [41].

The periodontitis stage was determined according to the criteria described by Tonetti et al. [42].

Samples were collected as previously described [43], and histopathological assessment confirmed 30 benign lesions, 30 OPMDs, and 30 OSCCs.

### 2.2. DNA Extraction and Microorganisms’ Detection, RNA Extraction, and Relative Gene Expression Analysis

Tumor tissue obtained from formalin-fixed, paraffin-embedded (FFPE) specimens was sectioned at 4 µm thickness using a microtome (Leica RM2245, Nussloch, Germany). Following deparaffinization in xylene, DNA was extracted from the tumor tissue using the phenol–chloroform method. Quantitative polymerase chain reaction (qPCR) was performed using SsoAdvanced™ Universal SYBR^®^ Green Supermix (Bio-Rad Laboratories, Hercules, CA, USA) to detect and quantify microorganisms. The qPCR reaction mixture in a final volume of 15 µL contained 20 ng of DNA template, 0.25 µM of each primer, and nuclease-free water. The thermal cycling conditions were as follows: initial denaturation at 95 °C for 10 min, followed by 45 cycles of 95 °C for 15 s and 58 °C for 30 s. Relative bacterial abundance was normalized to the human glyceraldehyde 3-phosphate dehydrogenase (GAPDH) reference gene using the 2^−ΔCt^ method. All reactions were performed in duplicate. No-template controls and positive controls (DNA extracted from a strain acquired from the American Type Culture Collection (ATCC), specifically *C. gingivalis* (ATCC33624)) were included in each qPCR run. Melting curve analysis was performed following amplification to verify the specificity of the PCR products.

RNA extraction, reverse transcription, and qPCR analysis of relative gene expression were performed as described in a previous study [43]. The primers used in this study are listed in Table 1.

### 2.3. Statistical Analysis

Statistical analyses were conducted using the Statistical Package for Social Sciences (SPSS, version 30.0; SPSS Inc., Chicago, IL, USA) and GraphPad Prism 9.0.00 (GraphPad Software, San Diego, CA, USA). Categorical data were analyzed utilizing Pearson’s χ^2^ test or Fisher’s exact test, depending on the sample size. Descriptive data were given as mean ± standard deviation and median values. The Kolmogorov–Smirnov test was applied to assess the distribution of continuous variables. The Kruskal–Wallis H test was used for comparisons among all three study groups, while pairwise comparisons were performed using the Mann–Whitney U test. Correlations between bacterial load and gene expression levels were evaluated using Spearman’s rank correlation coefficient.

## 3. Results

### 3.1. Demographic and Clinical Characteristics of the Study Population

The benign group included 12 papillomas, nine fibromas, and the same number of frictional keratoses. The OPMD group consisted of 17 leukoplakias, 5 erythroplakias, and 8 sublingual keratoses. Out of 30 OSCCs, four were subcategorized as verrucous oral squamous cell carcinomas. Statistically significant differences were observed for patients’ gender, periodontal stage, biofilm presence, and bleeding on probing between examined groups (Table 2). Plaque index (PI) and bleeding on probing (BOP) showed statistically significant differences: BP between OSCCs and benign lesions (*p* < 0.001) and OPMDs and benign lesions (*p* = 0.016); BOP between OSCCs and benign lesions (*p* < 0.001) and OSCCs and OPMDs (*p* = 0.035). Periodontal stage was significantly different between the OSCC group and the other two groups (*p* = 0.004 for OPMDs and *p* < 0.001 for benign lesions). No differences were found for other data (Table 2).

### 3.2. Bacterial Detection and Quantification Across Lesion Groups

*C. gingivalis* was detected in 2 samples from benign lesions, 11 OPMD, and 18 OSCC samples. Intergroup comparison showed an overall statistically significant difference (chi-square, *p* < 0.001), while pairwise comparisons did not reveal a significant difference between the OPMD and OSCC groups (Fisher’s exact test, *p* = 0.120).

**Figure 1:**
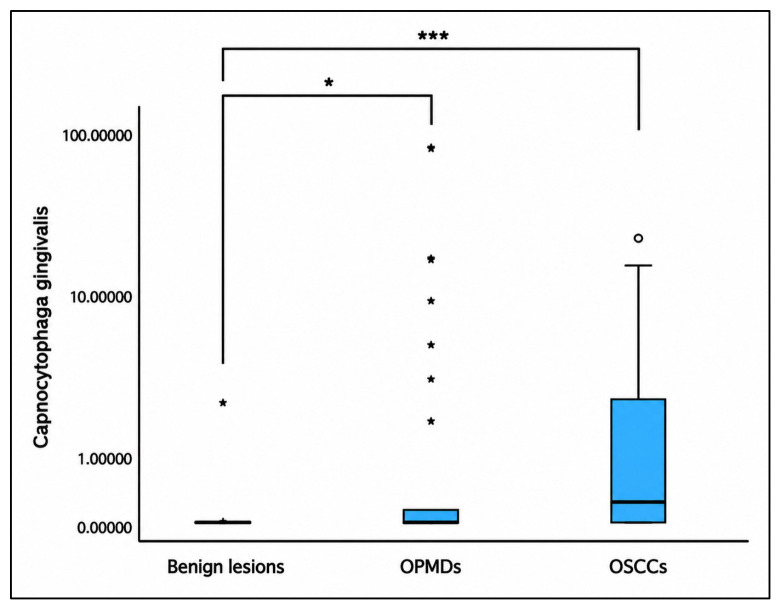
Boxplot diagram of bacterial quantification regardless of positivity between benign lesions, oral potentially malignant disorders (OPMDs), and oral squamous cell carcinomas (OSCCs). * *p* < 0.05, *** *p* < 0.001.

When bacterial quantification was analyzed in only positive samples, no statistically significant difference was observed (*p* = 0.662, Table 3). However, when all samples were included regardless of positivity, the difference was statistically significant (*p* = 0.001, Table 4). In pairwise comparisons, significant differences were observed between benign and OPMD lesions (Mann–Whitney, *p* = 0.013) and between benign and OSCC lesions (Mann–Whitney, *p* = 0.001), while the difference between OPMD and OSCC did not reach statistical significance (Mann–Whitney, *p* = 0.073) (Figure 1).

### 3.3. Gene Expression Analysis Across Lesion Groups

Gene expression patterns revealed statistically significant differences between examined groups for *PIK3CA* (*p* = 0.001) and *MAPK14* genes (*p* = 0.016).

**Figure 2:**
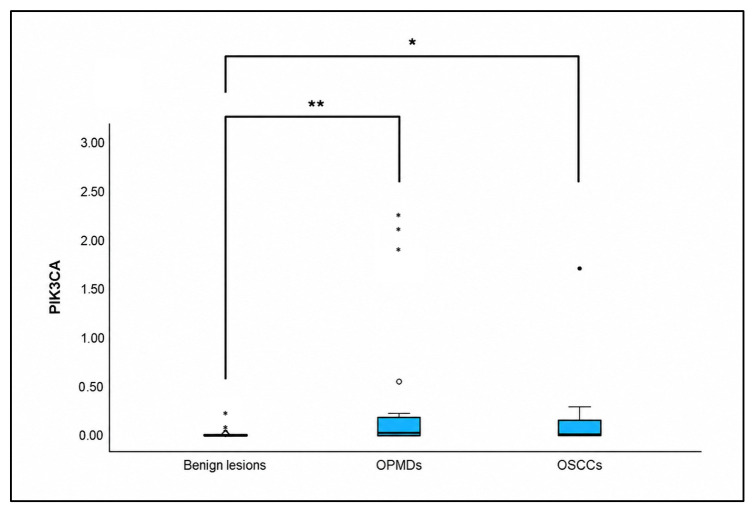
Expression levels of *PIK3CA* across benign, oral potentially malignant disorder (OPMD), and oral squamous cell carcinoma (OSCC) groups. * *p* < 0.05, ** *p* < 0.001.

Pairwise comparisons showed that for *PIK3CA*, significant differences were observed between benign and OSCC (*p* = 0.003; Bonferroni-adjusted, *p* = 0.010) and between benign and OPMD (*p* = 0.001; Bonferroni-adjusted, *p* = 0.002), while no significant difference was found between OSCC and OPMD (*p* = 0.639; Bonferroni-adjusted, *p* = 1.000) (Figure 2).

**Figure 3:**
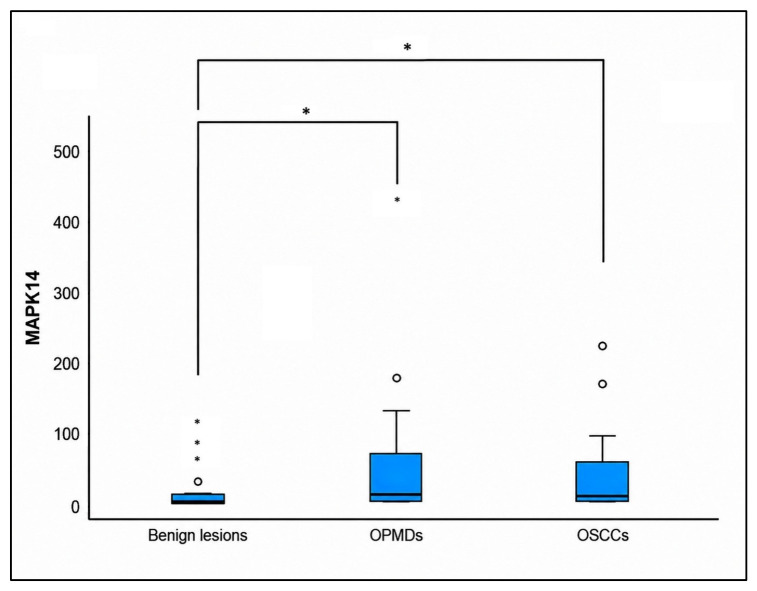
Expression levels of *MAPK14* across benign, oral potentially malignant disorder (OPMD), and oral squamous cell carcinoma (OSCC) groups. * *p* < 0.05.

Similarly, for *MAPK14*, significant differences were detected between benign and OSCC (*p* = 0.012; Bonferroni-adjusted, *p* = 0.036) and between benign and OPMD (*p* = 0.013; Bonferroni-adjusted, *p* = 0.039), whereas no significant difference was observed between OSCC and OPMD (*p* = 0.972; Bonferroni-adjusted, *p* = 1.000) (Figure 3).

### 3.4. Correlation Analysis

Correlation analyses were performed to explore the relationship between *C. gingivalis* abundance and gene expression levels. Among all *C. gingivalis*-positive samples, Spearman correlation analysis was used to assess the association between bacterial quantity and relative expression of investigated genes. However, no statistically significant correlations were observed.

Additionally, a separate correlation analysis was conducted within *C. gingivalis*-positive OSCC samples to evaluate these relationships within malignant lesions, revealing a significant positive correlation only for *VEGF* (ρ = 0.635, *p* = 0.005). To further explore the relationship, correlation analysis was additionally performed using all OSCC samples regardless of *C. gingivalis* positivity, revealing a positive correlation for relative gene expression of *Cyclin D1* (ρ = 0.435, *p* = 0.016) and *VEGF* (ρ = 0.529, *p* = 0.003). Regardless of *C. gingivalis* positivity, no significant correlations were found within the benign and OPMD groups.

## 4. Discussion

A growing body of epidemiological and clinical evidence has established an unequivocal association between poor oral hygiene and the development of head and neck cancers. Multiple studies have demonstrated that inadequate oral care contributes to chronic inflammation, microbial dysbiosis, and the accumulation of carcinogenic byproducts, all of which are implicated in carcinogenesis [44,45]. Literature data also clearly indicate a higher prevalence of male patients diagnosed with oral squamous cell carcinoma compared to females [46,47,48,49], which is in line with our findings. Analysis of the plaque index results revealed statistically significant differences, most notably between patients with OSCC and those with benign lesions. Although periodontal disease was present in all participants, bleeding on probing also demonstrated a statistically significant difference between the OSCC group and the benign lesion group, further underscoring the differential oral health status across these cohorts. Our results are in concordance with the results of a recent systematic review and meta-analysis [50]. An important aspect of the present study design is that all participants had periodontitis. This approach was intentionally adopted to minimize variability related to periodontal status and to enable a more direct comparison among benign lesions, OPMDs, and OSCCs within a relatively homogeneous periodontal background. However, this design does not allow the independent effects of periodontitis and OSCC on bacterial abundance or host gene expression to be distinguished.

The identification of periodontopathogens within oral mucosal lesions indicates a potential link to periodontal disease, reinforcing the hypothesis that microbial colonization may play a role in lesion development and progression. The true extent of this impact, however, is more accurately assessed when the microorganisms are present in substantial quantities within the lesion itself. Using qPCR analysis, a significant difference in abundance of *C. gingivalis* was observed between oral squamous cell carcinomas and benign lesions, as well as between potentially malignant lesions and benign lesions when all samples were considered. This combined evaluation of prevalence and bacterial load reveals a progressive increase in overall bacterial burden from benign lesions to OSCCs, reflecting both heightened colonization frequency and greater bacterial accumulation. The findings suggest that the evolving tumor microenvironment progressively fosters conditions that are more permissive for bacterial persistence and growth.

To the best of our knowledge, this is the first study dealing with the quantification of *C. gingivalis* across different lesions using the qPCR method. Previous studies have primarily been based on 16S rRNA sequencing [51,52], while the study by Zhu et al. [18] used fluorescence in situ hybridization assay (FISH) to demonstrate the abundance of *C. gingivalis* in OSCC samples. Our findings, which demonstrate a higher abundance of the bacterium in oral potentially malignant lesions compared to benign lesions, are particularly noteworthy and point toward a possible role in malignant transformation. Previous research has linked this bacterium to oral lichen planus [53], which was not the subject of research in this study.

The present study also established significantly elevated expression of *PIK3CA* and *MAPK14* in OSCCs and OPMDs compared to benign lesions. These findings support the involvement of key intracellular signaling pathways in both early and advanced stages of oral carcinogenesis. PIK3CA encodes the catalytic subunit of phosphatidylinositol 3-kinase (*PI3K*), a central regulator of cell proliferation, metabolism, and survival [54]. Mutations in *PIK3CA* or its increased expression have been associated with boosted tumor growth, greater resistance to apoptosis, and poor clinical outcomes [55]. *MAPK14* encodes the p38α MAP kinase, a stress-activated protein involved in cell differentiation, programmed cell death, inflammation, and response to environmental stimuli [56]. The simultaneous upregulation of *PIK3CA* and *MAPK14* points to the interplay between proliferative and stress-response pathways in oral cancer development. Crosstalk between the *PI3K/AKT* and *MAPK* pathways has been well recognized, with both contributing to tumor cell growth, angiogenesis, and resistance to therapy. Their higher expression in OPMD highlights the molecular continuity between premalignant and malignant states and supports the concept that these lesions carry substantial oncogenic potential at the molecular level even before histopathological progression is evident. However, it should be noted that gene expression patterns at the mRNA level may not directly correspond to pathway activation, which ultimately depends on protein synthesis and post-translational modifications, especially phosphorylation. Therefore, complementary approaches, particularly proteomic profiling and functional assays, are essential to confirm the biological activity of these pathways.

A positive correlation was observed in OSCC samples between *C. gingivalis* abundance and levels of *VEGF*, a key mediator in tumor angiogenesis, that drives neovascularization essential for tumor growth and metastatic potential [57]. The observed association between *C. gingivalis* abundance and *VEGF* expression should be interpreted with caution. Because the present study was observational, these findings do not demonstrate that *C. gingivalis* directly modulates *VEGF* or other signaling pathways. Rather, both bacterial abundance and host gene expression may reflect the broader inflammatory and polymicrobial tumor microenvironment. One plausible explanation is that *C. gingivalis*, as a member of the oral microbiota associated with periodontal disease, contributes to a pro-inflammatory microenvironment. Chronic inflammation is recognized as a driver of carcinogenesis through the activation of signaling pathways, which in turn can upregulate *VEGF* expression, thus promoting angiogenesis [58]. Correlation analysis across all OSCC samples irrespective of *C. gingivalis* status revealed a positive correlation for *Cyclin D1* and *VEGF* genes. This approach captures the combined influence of bacterial presence and abundance and was considered exploratory in nature. Moreover, because the overall microbial composition was not characterized, the potential contribution of other oral microorganisms cannot be excluded. Consequently, the observed correlations should be considered hypothesis-generating rather than evidence of a causal relationship.

Several limitations of this study should be acknowledged. First, the cross-sectional design allows the identification of associations but inherently lacks the capacity to establish causal relationships. Second, the modest sample size and the single-center design may limit the extent to which these findings can be generalized. Third, because all participants had periodontitis, the effects of periodontitis and those attributable to OSCC could not be distinguished. Furthermore, comprehensive microbiome profiling was not performed, preventing assessment of the contribution of other oral microorganisms. Finally, no functional or mechanistic experiments were performed to verify whether the observed associations between *C. gingivalis* abundance and host gene expression reflect direct biological effects. Future multicenter investigations with larger patient cohorts, integrated microbiome profiling, and mechanistic experimental models will be essential to validate and extend these findings.

## 5. Conclusions

The present study revealed a progressive rise in both the prevalence and overall burden of *C. gingivalis* across benign lesions, OPMDs, and OSCCs. Moreover, the observed association between bacterial load and *VEGF* gene expression, together with the increased expression of *PIK3CA* and *MAPK14*, particularly within the OSCC group, underscores the potential biological significance of *C. gingivalis* in the progression of oral squamous cell carcinoma.

## Figures and Tables

**Table 1:** List of primers used in the study.

Microorganism/Gene	Primer and/or Probe Sequence
***C. gingivalis***	Fwd: CTATCACCGACACCCGCAACCCAG Rv: GTCCACGCTCTATACACTCCTCCAC
***VEGF***	Fwd: GGGAGCTTCAGGACATTGCT Rv: GGCAACTCAGAAGCAGGTGA
***Cyclin D1***	Fwd: CGGAGGAGAACAAACAGATC Rv: GGGTGTGCAAGCCAGGTCCA
***PIK3CA***	Fwd: TTACCCTCTTCTGCCGGAGG Rv: AAGTGGATGCCCCACAGTTC
***DUSP16***	Fwd: AGGTGGGTTTGCTGAGTTCTC Rv: CTCGGGGATAAAGTCAGGCTT
***mTOR***	Fwd: GCCGCGCGAATATTAAAGGAA Rv: TGGTTTCCTCATTCCGGCTC
***MAPK14***	Fwd: ACTGGCTCGGCACACAGATG Rv: TCCCACTGACCAAATATCAACTG
***GAPDH***	Fwd: ATGGGGAAGGTGAAGGTCG Rv: GGGGTCATTGATGGCAACAATA

**Table 2:** Patients’ demographic and periodontal data results.

	Benign (*n* = 30)	OPMD (*n* = 30)	OSCC (*n* = 30)	*p* Value
**Age (mean ± SD); median)**	57.7 ± 13.4; 57.5	54.2 ± 15.7; 51	59.5 ± 12.7; 64	0.342
**sex (male/female)**	8/22	16/14	17/13	**0.038**
**smoking (yes/used to smoke/no)**	10/11/9	10/14/6	12/10/8	0.699
**alcohol (yes/used to drink/no)**	23/3/4	20/2/8	20/4/6	0.192
**periodontal stage (1/2/3/4)**	1/15/10/4	1/13/11/5	0/6/9/15	**0.002**
**periodontal probing depth (mm) (mean ± SD, median)**	3.2 ± 0.8; 3.1	3.2 ± 0.9; 3.4	3.4 ± 1.1; 3.2	0.564
**clinical attachment level (mm) (mean ± SD, median)**	3.6 ± 1.1; 3.6	3.8 ± 1.6; 3.7	4.4 ± 2.2; 4.1	0.453
**plaque index (%) (mean ± SD, median)**	53.5 ± 29.7; 47.5	70.2 ± 32.1; 78	80.6 ± 22.2; 87.5	**0.001**
**bleeding on probing (%) (mean ± SD, median)**	53.9 ± 26.6; 49	67 ± 31.6; 69	82.8 ± 18.3; 85	**0.001**

OPMD—oral potentially malignant disorders; OSCC—oral squamous cell carcinoma; SD—standard deviation. Significant at *p *< 0.05 (bold font).

**Table 3:** Comparative analysis of *C. gingivalis* quantification across positive samples within groups.

*C. gingivalis*	Benign Lesions	OPMDs	OSCCs
AM ± SD	1.2563 ± 1.7639	13.1290 ± 24.7835	7.5408 ± 14.6362
Med (IQR)	1.2563 (0.009–2.503)	3.5259 (0.1021–14.605)	2.0409 (0.4295–7.2702)
Kw test	*p* = 0.662		

AM—arithmetic mean; SD—standard deviation; Med—median; IQR—interquartile range; Kw—Kruskal-Wallis test.

**Table 4:** Comparative analysis of *C. gingivalis* quantification across all samples regardless of positivity.

*C. gingivalis*	Benign Lesions	OPMDs	OSCCs
AM ± SD	0.0838 ± 0.4570	4.8140 ± 15.9126	4.5245 ± 11.8193
Med (IQR)	0 (0–0)	0 (0–0.1395)	0.2596 (0–2.5775)
Kw test	***p* = 0.001**		

AM—arithmetic mean; SD—standard deviation; Med—median; IQR—interquartile range; Kw—Kruskal-Wallis test. Significant at *p *< 0.05 (bold font).

## Data Availability

The data presented in this study are available from the corresponding author upon reasonable request.
